# Higher fraction of inspired oxygen during anesthesia increase the risk of postoperative pulmonary complications in patients undergoing non-cardiothoracic surgery: a retrospective cohort study

**DOI:** 10.3389/fphys.2024.1471454

**Published:** 2024-10-18

**Authors:** Tianzhu Wang, Weixing Zhao, Libin Ma, Jing Wu, Xiaojing Ma, Luyu Liu, Jiangbei Cao, Jingsheng Lou, Weidong Mi, Changsheng Zhang

**Affiliations:** ^1^ Department of Anesthesia, First Medical Centre of Chinese PLA General Hospital, Beijing, China; ^2^ National Clinical Research Center for Geriatric Diseases, Chinese PLA General Hospital, Beijing, China

**Keywords:** lung-protective ventilation, fraction of inspired oxygen, postoperative pulmonary complications, elderly, non-cardiothoracic surgery

## Abstract

**Objective:**

The ideal intra-operative inspired oxygen concentration remains controversial. We aimed to investigate the association between the intraoperative fraction of inspired oxygen (FiO_2_) and the incidence of postoperative pulmonary complications (PPCs) in patients undergoing non-cardiothoracic surgery.

**Methods:**

This was a retrospective cohort study of elderly patients who underwent non-cardiothoracic surgery between April 2020 and January 2022. According to intraoperative FiO_2_, patients were divided into low (≤60%) and high (>60%) FiO_2_ groups. The primary outcome was the incidence of a composite of pulmonary complications (PPCs) within the first seven postoperative days. Propensity score matching (PSM) and inverse probability treatment weighting (IPTW) were conducted to adjust for baseline characteristic differences between the two groups. Multivariate logistic regression analysis was used to calculate the odds ratios (OR) for FiO_2_ and PPCs.

**Results:**

Among the 3,515 included patients with a median age of 70 years (interquartile range: 68–74), 492 (14%) experienced PPCs within the first 7 postoperative days. Elevated FiO_2_ was associated with an increased risk of PPCs in all the logistic regression models. The OR of the FiO_2_ > 60% group was 1.252 (95%CI, 1.015–1.551, P = 0.038) in the univariate analysis. In the multivariate logistic regression models, the ORs of the FiO_2_ > 60% group were 1.259 (Model 2), 1.314 (Model 3), and 1.32 (model 4). A balanced covariate distribution between the two groups was created using PSM or IPTW. The correlation between elevated FiO_2_ and an increased risk of PPCs remained statistically significant with PSM analysis (OR, 1.393; 95% CI, 1.077–1.804; P = 0.012) and IPTW analysis (OR, 1.266; 95% CI, 1.086–1.476; P = 0.003).

**Conclusion:**

High intraoperative FiO_2_ (>60%) was associated with the postoperative occurrence of pulmonary complications, independent of predefined risk factors, in elderly non-cardiothoracic surgery patients. High intraoperative FiO_2_ should be applied cautiously in surgical patients vulnerable to PPCs.

## 1 Introduction

Postoperative pulmonary complications (PPCs), such as respiratory infection, atelectasis, and hypoxemia, occurred in 10%–59% of patients undergoing surgery, especially in elderly patients with physiological dysfunction. The occurrence of PPCs is associated with an increased risk of morbidity and mortality in hospitalized patients (Pearse et al., 2012; Canet et al., 2010; Fernandez-Bustamante et al., 2017).

Several risk factors are closely related to PPCs, including patient factors, surgical types and timing, and anesthesia management (Sun et al., 2023). Among these factors, the benefits and risks of a high fraction of inspired oxygen (FiO_2_) during anesthesia remain debated in the scientific literature. The World Health Organization (WHO) recommends applying high FiO_2_ to reduce the risk of postoperative surgical site infections in patients undergoing general anesthesia (Leaper and Edmiston, 2017). Chinese anesthesiologists have become accustomed to administering higher FiO_2_ levels throughout the course of intraoperative mechanical ventilation management (Wang et al., 2022). On one hand, high intraoperative FiO_2_ could increase the safety margin in cases of intraoperative emergencies, such as desaturation in cases of airway loss or ventilation failure (Weenink et al., 2020). However, it may accelerate atelectasis formation during mechanical ventilation, promote coronary vasoconstriction, increase peripheral vascular resistance, and decreases cardiac output (Martin and Grocott, 2015).

Although some recent studies have analyzed the correlation between high FiO_2_ and PPCs, the recruited samples were not large enough to be representative. In addition, no articles have specifically analyzed elderly patients undergoing non-cardiothoracic surgery. Therefore, we conducted a retrospective study to examine the association between the level of FiO_2_ and the incidence of PPCs in patients undergoing non-cardiothoracic surgery.

## 2 Materials and methods

The methods used in this study were modified based on our team’s previous publication (Zhang et al., 2021). The study protocol was reviewed and approved by the Institutional Ethics Committee of the Chinese PLA General Hospital (No. S2021-135-01). The study protocol complied with the Declaration of Helsinki, and the need for informed consent was waived because the study was retrospective. This manuscript adhered to the applicable guidelines as presented in the Strengthening the Reporting of Observational Studies in Epidemiology (STROBE) guidelines.

### 2.1 Study subjects

We identified patients who had undergone non-cardiothoracic surgery between April 2020 and January 2022 at the First Medical Center of the Chinese PLA General Hospital, a tertiary academic hospital in Beijing, China.

**FIGURE 1:**
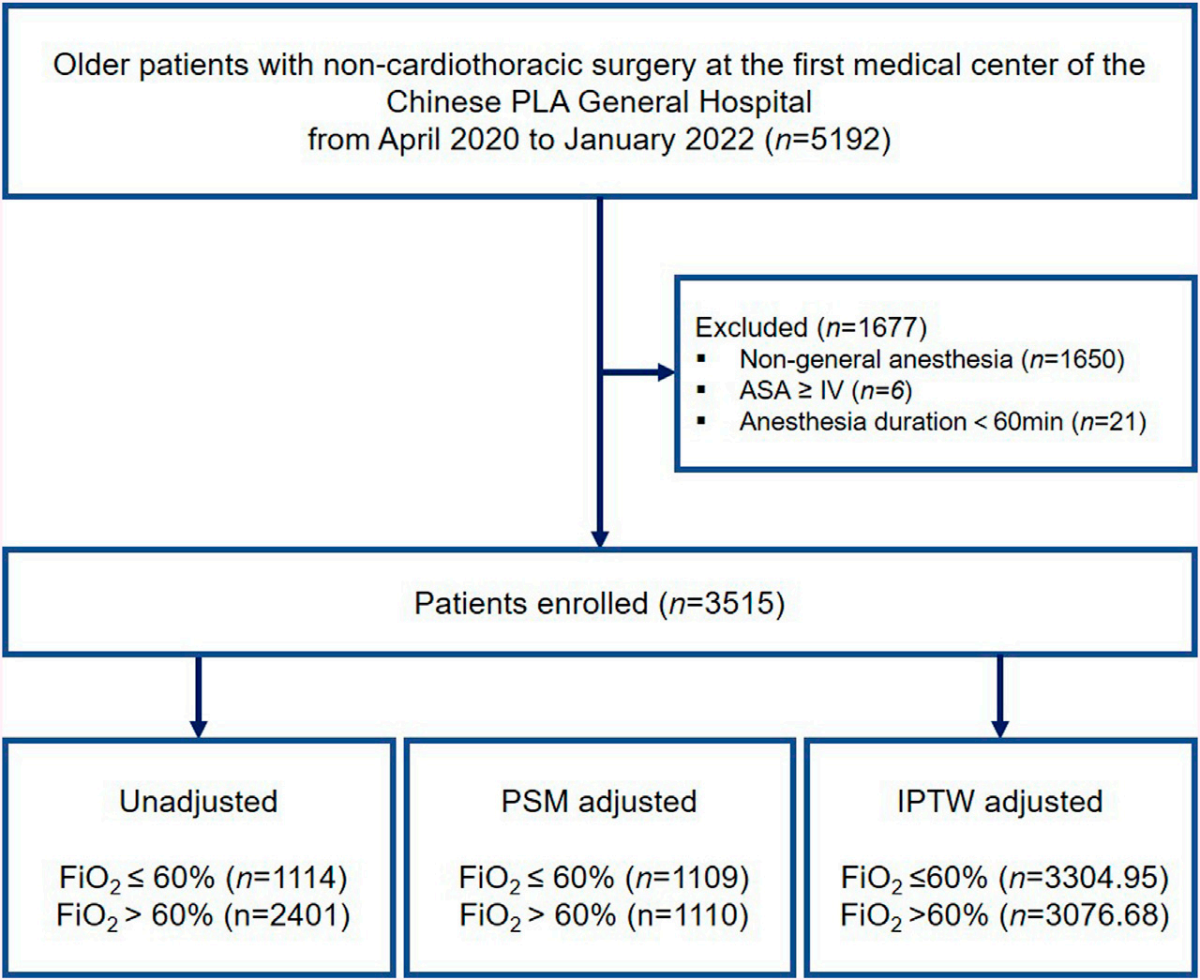
Study flow diagram. ASA, American Society of Anesthesiologists; PSM, propensity score matching; IPTW, inverse probability treatment weighting.

The inclusion criteria were as follows: age 65 years or older; BMI >18 kg/m^2^ and <35 kg/m^2^; and duration of general anesthesia >60 min. Patients who presented with an American Society of Anesthesiologists (ASA) classification ≥ IV or had missing clinical data of >50% were excluded. Among the patients who underwent multiple surgeries during the study period, only the first eligible surgery was considered. A flow diagram of the patient selection process is shown in Figure 1.

### 2.2 Study outcome

The primary outcome was the incidence of a composite of pulmonary complications within the first seven postoperative days. PPCs were defined using the Assess Respiratory Risk in Surgical Patients in Catalonia (ARISCAT) study criteria, including postoperative respiratory infection, respiratory failure, bronchospasm, atelectasis, pulmonary embolism, pleural effusion, aspiration pneumonia, pulmonary edema, and pneumothorax (Canet et al., 2010; Mazo et al., 2014).

### 2.3 Definition of variables and data collection

After descriptive analysis of the retrieved data, we found that a large number of physicians in our department prefer to apply a high intraoperative FiO_2_, while others tend to use a FiO_2_ of less than 60%. Only 2.22% of the patients received a FiO_2_ of 21%–50% intraoperatively, 29.47% received a FiO_2_ of 50%–60%, 7.1% received a FiO_2_ of 60%–90% and 61.22% received a FiO_2_ of 100%. The first quartile (Q1) of the distribution was at an FiO_2_ of 60%. Therefore, according to the distribution of FiO_2_ in the retrieved data, the threshold of FiO_2_ in this study was set to 60% for the prediction of PPCs. Consequently, the FiO_2_ was stratified into low (≤60%) and high (>60%) for subsequent analyses.

The electronic medical record system was used to collect demographic, preoperative and postoperative data including medication, lab results and radiology reports, etc. Preoperative covariates of interest, such as age, sex, body mass index (BMI), ASA classification, hypertension, diabetes mellitus, smoking history, drinking history, and tumor characteristics (benign or malignant), were noted. The indices derived from the preoperative laboratory data, including hemoglobin, leukocyte, creatinine, and glucose levels, were defined as the most recent counts measured within 3 days prior to surgery. In addition, surgical and anesthesia information was extracted from the anesthesia information management system, including tidal volume, respiratory rate, surgery type (laparoscopic or open surgery), duration of anesthesia, blood loss, fluid loss, infusion volume, crystalloid or colloid infusion volume, blood transfusion volume, intraoperative antibiotics, and opioid use. All related data were extracted from the database to calibrate the results of PPCs. Data were manually revised and reviewed according to the original medical and anesthesia records, if abnormal, such as missing, duplicate and outlier, were found during the data cleansing process.

### 2.4 Statistical analysis

The baseline characteristics and outcomes of the patients were summarized using frequencies and descriptive statistics. Continuous variables are expressed as mean (SD) or median (interquartile range), and categorical variables are presented as n (%). In the analysis of baseline data, the chi-square test was used for classified data, and Fisher’s test was used if the frequency was <5. If the continuous data were normal, variance analysis was used. If continuous data were not normal, a rank sum test was used.

Multivariate logistic regression was performed to control for potential confounding effects and evaluate the relationship between FiO_2_ and PPCs. In the logistic regression analysis, multiple models were constructed with different covariates to calculate the odds ratios (OR) of FiO_2_ and PPCs.

In subsequent analyses, propensity score (PS) analysis was conducted, including propensity score matching (PSM) and inverse probability treatment weighting (IPTW), to examine the PPCs associated with FiO_2_. To adjust for between-group differences, PSs were developed to reflect the application of FiO_2_ in each patient during surgery. PS, a composite score, was derived from synthesized baseline characteristics. Clinically relevant covariates (the aforementioned 25 covariates) were included in the multivariate logistic regression model to yield PS. In PSM, matching between the two groups was randomly conducted with PS at a 1:2 ratio using the greedy nearest-neighbor approach, with a caliper width of 0.2. Symmetric trimming was performed in IPTW to minimize the adverse effects of extreme PS outliers. Patients with an estimated PS beyond the range (10%–90%) were excluded. After obtaining matched or weighted data, kernel density plots and standardized mean difference (SMD) were applied to assess the balance of covariates between the two groups. An SMD <0.1 was deemed as acceptable deviations for the particular covariate. The association between FIO_2_ and PPCs was estimated using multivariate logistic regression analysis to calculate the adjusted OR.

In several previous meta-analyses or studies, advanced age, duration of anesthesia, laparoscopic surgery, smoking history, and BMI were associated with shortened PPCs. Therefore, subgroup analyses were performed according to the aforementioned parameters to explore the potential interactions. Multivariate logistic regression analyses were performed separately for each subgroup to calculate the adjusted OR. A two-sided P < 0.05 was considered statistically significant. Statistical analyses were performed using R (version 4.0.5; R Foundation for Statistical Computing, Vienna, Austria).

## 3 Results

### 3.1 Patient characteristics

A total of 5,192 elderly patients who underwent non-cardiothoracic surgery between April 2020 and January 2022 were enrolled in this study. Figure 1 illustrates a flow diagram of patient selection. After applying the inclusion and exclusion criteria, 3,515 eligible patients remained in the analysis, with a median age of 70 years (IQR: 68–74 years), of whom 1,618 (46.0%) were women. Among the patients, 1896 (53.94%) underwent laparoscopic surgery and 303 (0.086%) received blood transfusions. Of the entire patient cohort, 492 (14.00%) patients experienced PPCs after surgery.

Patients were then grouped into low (≤60%, n = 1,114, 31.69%) and high (>60%, n = 2,401, 68.31%) FiO_2_ groups. The differences in baseline characteristics between the groups are shown in Table 1. Compared with patients with FiO_2_ ≤ 60%, those with FiO_2_ > 60% had a higher incidence of PPCs.

**TABLE 1:** Baseline characteristics unadjusted sample, propensity score-matched sample, and inverse probability of treatment-weighted sample.

Characteristic		Unadjusted sample (N = 3,515)			PSM adjusted (N = 2,219)			IPTW adjusted (N = 6,381.63)		
		FiO_2_ ≤ 60% (n = 1,114)	FiO_2_ > 60% (n = 2,401)	SMD	FiO_2_ ≤ 60% (n = 1,109)	FiO_2_ > 60% (n = 1,110)	SMD	FiO_2_ ≤ 60% (n = 3,304.95)	FiO_2_ > 60% (n = 3,076.68)	SMD
Age (median [IQR])		70.00 [68.00, 74.00]	71.00 [68.00, 74.00]	0.016	70.00 [68.00, 74.00]	71.00 [68.00, 74.00]	0.049	70.00 [68.00, 74.00]	71.00 [68.00, 74.00]	0.011
Sex (%)	Male	597 (53.6)	1,300 (54.1)	0.011	595 (53.7)	612 (55.1)	0.03	1,752.7 (53.0)	1,648.2 (53.6)	0.011
	Female	517 (46.4)	1,101 (45.9)		514 (46.3)	498 (44.9)		1,552.3 (47.0)	1,428.5 (46.4)	
BMI (median [IQR])		24.25 [22.49, 26.62]	24.80 [22.66, 26.91]	0.087	24.27 [22.50, 26.62]	24.49 [22.49, 26.43]	0.028	24.34 [22.60, 26.67]	24.56 [22.49, 26.68]	0.02
ASA classification (%)	Ⅰ	5 (0.4)	27 (1.1)	0.078	5 (0.5)	12 (1.1)	0.073	14.4 (0.4)	39.2 (1.3)	0.091
	Ⅱ	891 (80.0)	1,922 (80.0)		887 (80.0)	886 (79.8)		2,644.9 (80.0)	2,448.3 (79.6)	
	Ⅲ	218 (19.6)	452 (18.8)		217 (19.6)	212 (19.1)		645.7 (19.5)	589.1 (19.1)	
Hypertension (%)	0	506 (45.4)	1,068 (44.5)	0.019	504 (45.4)	488 (44.0)	0.03	1,515.3 (45.8)	1,368.0 (44.5)	0.028
	1	608 (54.6)	1,333 (55.5)		605 (54.6)	622 (56.0)		1,789.7 (54.2)	1,708.7 (55.5)	
Diabetes mellitus (%)	0	790 (70.9)	1,721 (71.7)	0.017	786 (70.9)	792 (71.4)	0.011	2,348.9 (71.1)	2,207.3 (71.7)	0.015
	1	324 (29.1)	680 (28.3)		323 (29.1)	318 (28.6)		956.1 (28.9)	869.4 (28.3)	
Preoperative hemoglobin (median [IQR])		129.00 [119.00, 141.00]	132.00 [121.00, 142.00]	0.105	129.00 [119.00, 141.00]	130.00 [119.00, 140.00]	0.004	130.00 [120.00, 141.00]	130.00 [119.00, 140.00]	0.024
Preoperative leucocyte (median [IQR])		5.74 [4.82, 6.93]	5.82 [4.85, 7.03]	0.017	5.75 [4.82, 6.92]	5.80 [4.85, 7.04]	0.027	5.74 [4.82, 6.89]	5.83 [4.81, 7.06]	0.029
Preoperative serum creatinine (median [IQR])		72.55 [61.70, 83.77]	72.00 [62.20, 84.40]	0.037	72.60 [61.70, 83.80]	72.50 [62.70, 84.38]	0.046	72.16 [61.60, 83.35]	71.60 [61.70, 84.20]	0.037
Preoperative glucose (median [IQR])		5.27 [4.83, 6.04]	5.33 [4.84, 6.14]	0.048	5.27 [4.83, 6.04]	5.29 [4.80, 6.11]	0.011	5.27 [4.83, 6.03]	5.33 [4.83, 6.14]	0.05
History of smoking (%)	No	825 (74.1)	1,729 (72.0)	0.046	821 (74.0)	805 (72.5)	0.034	2,461.0 (74.5)	2,224.5 (72.3)	0.049
	Yes	289 (25.9)	672 (28.0)		288 (26.0)	305 (27.5)		843.9 (25.5)	852.2 (27.7)	
History of alcohol consumption (%)	No	851 (76.4)	1,754 (73.1)	0.077	846 (76.3)	817 (73.6)	0.062	2,515.2 (76.1)	2,267.6 (73.7)	0.055
	Yes	263 (23.6)	647 (26.9)		263 (23.7)	293 (26.4)		789.8 (23.9)	809.1 (26.3)	
Malignant tumor (%)	0	530 (47.6)	974 (40.6)	0.142	527 (47.5)	546 (49.2)	0.033	1,481.1 (44.8)	1,466.6 (47.7)	0.057
	1	584 (52.4)	1,427 (59.4)		582 (52.5)	564 (50.8)		1823.8 (55.2)	1,610.1 (52.3)	
Laparoscopic surgery (%)	0	558 (50.1)	1,061 (44.2)	0.118	556 (50.1)	529 (47.7)	0.05	1,627.4 (49.2)	1,441.4 (46.8)	0.048
	1	556 (49.9)	1,340 (55.8)		553 (49.9)	581 (52.3)		1,677.5 (50.8)	1,635.3 (53.2)	
Anesthesia duration (median [IQR])		215.00 [165.00, 265.00]	195.00 [150.00, 255.00]	0.18	215.00 [165.00, 265.00]	209.00 [160.00, 270.00]	0.01	209.31 [160.00, 260.00]	210.00 [160.00, 269.00]	0.052
Blood losses (median [IQR])		100.00 [50.00, 200.00]	50.00 [50.00, 200.00]	0.052	100.00 [50.00, 200.00]	100.00 [50.00, 200.00]	0.062	100.00 [50.00, 200.00]	100.00 [50.00, 200.00]	0.06
Crystalloid infusion (median [IQR])		1,600.00 [1,200.00, 2,100.00]	1,600.00 [1,100.00, 2,100.00]	0.176	1,600.00 [1,200.00, 2,100.00]	1,600.00 [1,100.00, 2,100.00]	0.085	1,600.00 [1,200.00, 2,100.00]	1,600.00 [1,100.00, 2,100.00]	0.043
Colloid infusion (median [IQR])		500.00 [500.00, 500.00]	500.00 [500.00, 500.00]	0.058	500.00 [500.00, 500.00]	500.00 [500.00, 500.00]	0.013	500.00 [500.00, 500.00]	500.00 [500.00, 500.00]	0.007
Blood transfusion ml (%)	0	1,002 (89.9)	2,210 (92.0)	0.073	998 (90.0)	992 (89.4)	0.02	3,001.1 (90.8)	2,774.7 (90.2)	0.021
	1	112 (10.1)	191 (8.0)		111 (10.0)	118 (10.6)		303.9 (9.2)	302.0 (9.8)	
Fluids lost amount (median [IQR])		300.00 [150.00, 650.00]	300.00 [100.00, 600.00]	0.121	300.00 [150.00, 650.00]	350.00 [150.00, 700.00]	0.003	300.00 [150.00, 600.00]	300.00 [150.00, 600.00]	0.008
Fluids infused amount (median [IQR])		2,100.00 [1,600.00, 2,617.50]	1,700.00 [1,208.00, 2,600.00]	0.199	2,100.00 [1,600.00, 2,625.00]	1,800.00 [1,300.00, 2,600.00]	0.067	2,050.00 [1,600.00, 2,600.00]	1,900.00 [1,500.00, 2,600.00]	0.03
Tidal volume (median [IQR])		450.00 [400.00, 450.00]	450.00 [400.00, 450.00]	0.107	450.00 [400.00, 450.00]	450.00 [400.00, 450.00]	0.002	450.00 [400.00, 450.00]	450.00 [400.00, 450.00]	0.063
Respiratory rate (median [IQR])		13.00 [12.00, 14.00]	13.00 [12.00, 14.00]	0.081	13.00 [12.00, 14.00]	13.00 [12.00, 14.00]	0.046	13.00 [12.00, 14.00]	13.00 [12.00, 14.00]	0.01
Intraoperative antibiotics (%)	0	175 (15.7)	314 (13.1)	0.075	173 (15.6)	153 (13.8)	0.051	516.8 (15.6)	396.3 (12.9)	0.079
	1	939 (84.3)	2,087 (86.9)		936 (84.4)	957 (86.2)		2,788.1 (84.4)	2,680.3 (87.1)	
Intraoperative opioid (%)	0	33 (3.0)	124 (5.2)	0.112	33 (3.0)	42 (3.8)	0.045	72.8 (2.2)	52.1 (1.7)	0.037
	1	1,081 (97.0)	2,277 (94.8)		1,076 (97.0)	1,068 (96.2)		3,232.1 (97.8)	3,024.6 (98.3)	
PPCs (%)	0	978 (87.8)	2,045 (85.2)	0.077	974 (87.8)	935 (84.2)	0.104	2,897.0 (87.7)	2,616.6 (85.0)	0.076
	1	136 (12.2)	356 (14.8)		135 (12.2)	175 (15.8)		407.9 (12.3)	460.1 (15.0)	

BMI, body mass index; ASA, american society of anesthesiologists.

### 3.2 Correlation between FiO_2_ and PPCs

Three logistic regression models were used to investigate the correlation between FiO_2_ and PPCs. Elevated FiO_2_ was associated with an increased risk of PPCs in all models. The OR of the FiO_2_ > 60% group was 1.252 (95%CI:1.015–1.551, P = 0.038) in the univariate analysis. In the multivariate logistic regression models, the ORs of the FiO_2_ > 60% group were 1.259 (model 2), 1.314 (model 3), and 1.32 (model 4), respectively. The P values were <0.05 for all models (Table 2). The overall univariate and multivariate logistic regression results are presented in Table 2.

**TABLE 2:** Association between intraoperative FiO_2_ and occurrence of PPCs using the logistic regression model and propensity score analysis.

Analyses method	OR	95% CI	P-value
Logistic regression analysis (n = 3,515)			
Model 1 (Univariate model)	1.252	1.015–1.551	0.04
Model 2 (Preoperative patient-related covariates adjusted)	1.259	1.014–1.572	0.04
Model 3 (Surgical and anesthetic covariates adjusted)	1.314	1.055–1.645	0.02
Model 4 (Fully adjusted)	1.320	1.055–1.660	0.02
Propensity score analysis (multivariate)			
Model PSM (n = 2,219)	1.393	1.077–1.804	0.01
Model IPTW (n = 6,381.63)	1.266	1.086–1.476	<0.01

PSM, propensity score matching; IPTW, inverse probability treatment weighting.

### 3.3 PSM and IPTW analysis and adjustment

**FIGURE 2:**
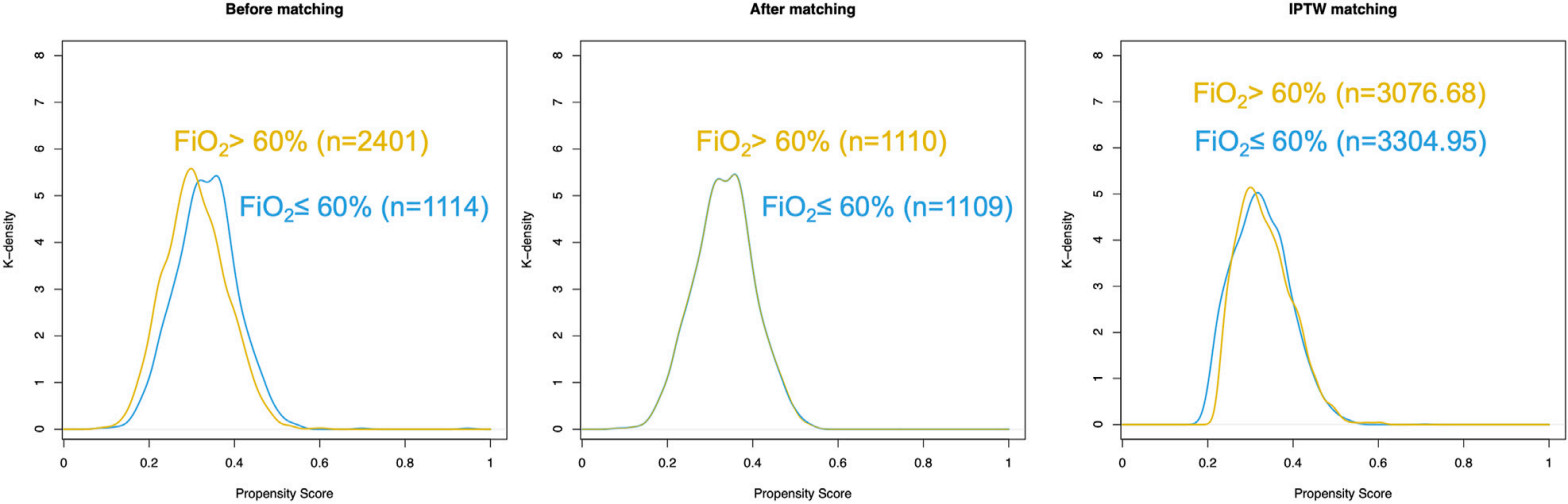
Distribution of propensity scores in patients with postoperative pulmonary complications (low and high FiO_2_ groups). **(A)** Before matching. **(B)** After matching. **(C)** IPTW matching.

**FIGURE 3:**
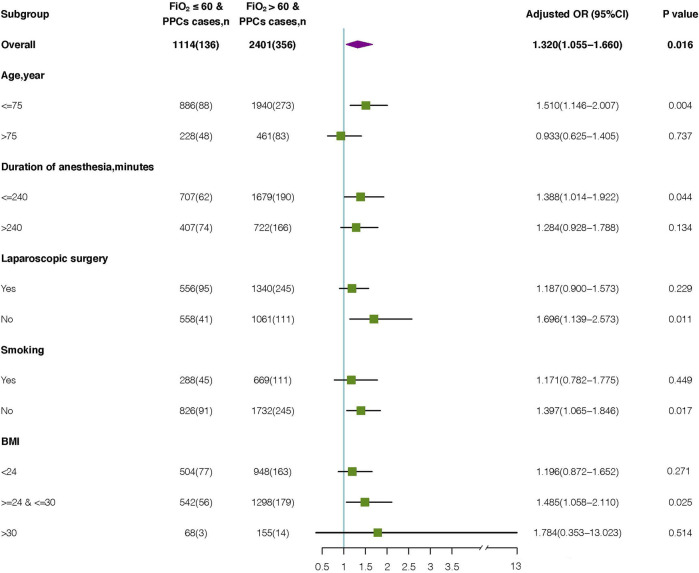
Subgroup analysis of the association between intraoperative FiO_2_ and occurrence of PPCs. OR, odds ratio.

PSM and IPTW analyses were performed as planned. Seven variables (BMI, preoperative hemoglobin level, anesthesia duration, malignant tumor, tidal volume, respiratory rate, and intraoperative opioid use) were matched in constructing the PSM cohort. Eight variables (BMI, sex, preoperative hemoglobin, anesthesia duration, malignant tumor, tidal volume, respiratory rate, and intraoperative opioid use) were matched to construct the IPTW cohort. A total of 1,114 patients in the FiO_2_ ≤ 60% group and 2,401 patients in the FiO_2_ > 60% group were matched. The distribution of propensity scores of the patients before and after PSM and IPTW is displayed in Figures 2, 3, respectively. The baseline characteristics and variables were balanced between the two groups (SMD <0.1) (Table 1). In the logistic regression analysis performed after PSM, FiO_2_ > 60% was still an independent predictor of PPCs, with an OR of 1.393 (95% CI: 1.077–1.804, P = 0.012). Similarly, in the logistic regression performed after IPTW, FiO_2_>60% was an independent predictor of PPCs, with an OR of 1.266 (95% CI: 1.086–1.476, P = 0.003) (Table 2).

### 3.4 Subgroup analyses

Among the 2,401 elderly patients with FiO_2_ > 60%, 461 (19.2%) were aged >75 years, 722 (30.1%) had a duration of anesthesia >240 min, 1,340 (55.8%) underwent laparoscopic surgery, 669 (27.9%) had a history of smoking, and 948 (39.5%) had 18<BMI<24 kg/m^2^. The BMI of 155 (6.5%) patients was 30–35 kg/m^2^.

Advanced age, duration of anesthesia, laparoscopic surgery, smoking history, and BMI were associated with shortened PPCs. To explore potential interactions, we performed a subgroup analysis according to the parameters above. (Figure 3). Multivariate logistic regression analyses were conducted for each subgroup to calculate the adjusted OR. The OR of FiO_2_ was significant for age subgroups [≥75 years: OR (95%CI): 0.933 (0.625–1.405), P = 0.737; <75 years: OR (95%CI): 1.510 (1.146–2.007), P = 0.004]. Additionally, an increased risk of PPCs was observed for both duration of anesthesia ≤240 min (odds ratio [OR], 1.388; 95% CI: 1.014–1.922; P = 0.044) and duration of anesthesia >240 min (OR: 1.284; 95% CI: 0.928–1.788; P = 0.134). In patients who underwent laparoscopic surgery, high FiO_2_ was significantly correlated with PPCs (OR, 1.187; 95%CI: 0.900–1.573; P = 0.229). This increased risk was also significant in those who underwent non-laparoscopic surgery (OR, 1.696; 95% CI: 1.139–2.573; P = 0.011). The correlation between Intraoperative FiO_2_ and PPCs was significant in individuals with (OR:1.171; 95%CI: 0.782–1.775; P = 0.449) and without (OR: 1.397; 95%CI: 1.065–1.846; P = 0.017) history of smoking. Additionally, in each BMI group, high FiO_2_ was significantly associated with an increased risk of PPCs (18<BMI<24 group: OR: 1.196; 95% CI: 0.872–1.652; P = 0.271) (24≤BMI≤30 group: OR: 1.148; 95% CI: 1.058–2.110; P = 0.025) (30<BMI<35 group: OR: 1.784; 95% CI: 0.353–13.023; P = 0.514).

## 4 Discussion

In this retrospective analysis of surgical patients undergoing intraoperative mechanical ventilation for elderly non-cardiothoracic surgery, we found that a high intraoperative FiO_2_ (>60%) was significantly associated with a composite outcome of PPCs. We conducted propensity score matching (PSM) and inverse probability treatment weighting (IPTW) to adjust for baseline characteristic differences between intraoperative high FiO_2_ and low FiO_2_ patients, and the findings remained consistent. However, this correlation existed only in non-laparoscopic surgery and was not significant in laparoscopic surgery in our subgroup analysis.

### 4.1 Controversy over FiO_2_

Although the World Health Organization (WHO) in 2018 and the Centers for Disease Control and Prevention (CDC) in 2017 recommended the application of high perioperative FiO_2_ (Berríos-Torres et al., 2017; World Health Organization, 2018), this recommendation and related meta-analyses used to support them have been widely criticized (de Jonge et al., 2019; Myles et al., 2019; Hedenstierna et al., 2019). The ideal FiO_2_ setting during intraoperative mechanical ventilation is a topic of ongoing debate among anesthesiologists. Advocates argue that high-inspired FiO_2_ reduces surgical site infection (SSI) (Hovaguimian et al., 2013; Kuh et al., 2023) and postoperative nausea and vomiting (PONV) (Greif et al., 1999) and extends the safety margin in cases of acute intraoperative emergencies. Conversely, opponents contend that high-inspired FiO_2_ does not reduce SSI and further increases atelectasis formation (Park et al., 2021), oxidative stress during surgery (Oldman et al., 2021), and exacerbates cancer effects (Meyhoff et al., 2012).

At present, studies on the effects of high intraoperative FiO_2_ on PPCs are conflicting. We found a significant association between high intraoperative FiO_2_ and PPCs, which is consistent with a large, single-center retrospective database study including 73,922 cases. High intraoperative FiO_2_ was associated with major respiratory complications in a dose-dependent manner (Staehr-Rye et al., 2017). Moreover, a multicenter observational cohort study showed that patients at the 75th percentile for the area under the curve of the FiO_2_ had 14% greater odds of lung injury (12%–16%) than patients at the 25th centile (McIlroy et al., 2022). However, in a randomized trial of 251 patients who underwent abdominal surgery with lung-protective ventilation, the incidence of PPCs was similar in patients who received FiO_2_ of 30% versus 80%, although the severity of PPCs was reduced by low FiO_2_ (Li et al., 2020). Likewise, in a post-hoc analysis of a prospective single-center alternating cohort trial including nearly 5,000 surgical patients who received 30% or 80% FiO_2_ during surgery, the incidence of PPCs was similar between the groups (Cohen et al., 2019). Similarly, Ferrando et al. found that the occurrence rate of PPCs did not differ between patients ventilated with FiO_2_ of 30% and 80%, despite the application of a protocolized ventilation strategy (Ferrando et al., 2020). Notably, the outcome measurements, evaluative dimensions, and ventilation strategies were not entirely the same among these studies and had a lower power of testing as a secondary outcome.

### 4.2 Underlying mechanisms of lung injury

Several mechanisms have been reported to contribute to lung injury after application of high FiO_2_ (Horncastle and Lumb, 2019). Higher FiO_2_ maintained after intubation promoted atelectasis in 90% of patients and was related to an increase in the low ventilation perfusion ratio (Östberg et al., 2017).-Atelectasis is likely to be the focus of infection and may contribute to additional pulmonary complications. One of the main reasons for the development of PPCs is hyperoxia-induced absorptive atelectasis, which is common during general anesthesia and can persist for several days after surgery (Hedenstierna and Edmark, 2010). Other plausible mechanisms include the generation of reactive oxygen species (ROS) and pro-inflammatory factors and the impairment of gas exchange (Nagato et al., 2012; Romagnoli et al., 2015; Gore et al., 2010). Considering that hyperoxia in cardiopulmonary bypass (CPB) did not result in any increase in respiratory complications because of non-ventilated lung during CPB (Abou-Arab et al., 2019), oxygen toxicity to the lung may occur directly through the endotracheal tube to the lung.

### 4.3 Subgroup analyses

In subgroup analysis, we found that in patients aged ≤75 years, duration of anesthesia ≤240 min, non-laparoscopic surgery, non-smoking, and 24≤ BMI ≤30 subgroup, high FiO_2_ was associated with an increase in PPCs. A systematic review showed that when compared with patients <50 years old, patients aged 70–79 years had odds ratios (OR) of 3.90 (CI: 2.70–5.65) of developing PPCs (Smetana et al., 2006). Therefore, the results showed that only patients in younger older (aged ≤75 years) subgroup, high FiO_2_ was associated with an increase in PPCs. In a meta-analysis of 107 cohort and case-control studies, preoperative smoking was associated with an increased risk for PPCs (RR 1.73, 95% CI: 1.35–2.23) (Gore et al., 2010). Likewise, only in non-smoking subgroup, high FiO_2_ was associated with an increase in PPCs. In a retrospective study (n = 141,802), PPCs were no more common among obese adults (BMI >30 kg/m^2^) than among those with a healthy weight (BMI 18.5–24.9 kg/m^2^) (Sood et al., 2015). Interestingly, underweight patients sustained more PPCs, which may be due to the experience of frailty. A recent study found that frailty was significantly associated with PPCs in elderly patients who underwent cardiac surgery (Fan et al., 2023; Chen et al., 2022). It is plausible that only in 24≤ BMI ≤30 subgroup, high FiO_2_ was associated with an increase in PPCs. Surgical procedures lasting more than 3–4 h are associated with a higher risk of pulmonary complications (McAlister et al., 2003). Similarly, only in duration of anesthesia ≤240 min subgroup, high FiO_2_ was associated with an increase in PPCs. In patients aged >75 years, duration of anesthesia >240 min, current smoking, BMI <24 kg/m^2^, and BMI >30 kg/m^2^ subgroups, high FiO_2_ was not associated with PPCs. It might be due to the fact that compared to advanced age, duration of surgery, smoking, and BMI, FiO_2_ had weaker impact on PPCs. In the laparoscopic surgery subgroup, patients who underwent laparoscopic surgery were more likely to develop atelectasis, and thus, PPCs were more common. Considering the differential power of the occurrence of PPCs, it is plausible that high FiO_2_ was not associated with PPCs in the laparoscopic surgery subgroup.

### 4.4 Strengths and limitations

The analyses in this study were based on a large dataset that largely reflects routine clinical practice. Data were retrieved from accurate prospective recordings of intraoperative management and postoperative complications. Before initiating the data analysis, we discussed and finalized the protocol, including definitions of risk factors and outcomes, statistical methods, and quality control. This observational design allowed us to collect a large number of 3,515 elderly surgical patients, which can provide sufficient power to detect differences in relatively infrequent complications. Meanwhile, a variety of potential confounding factors were included, such as preoperative and intraoperative data, which allowed for precise effect size evaluation. Additionally, sensitivity analyses, such as PSM or IPTW and subgroup analyses, were successfully performed to further validate the robustness of our findings. Finally, to the best of our knowledge, this is the first study to explore the association between intraoperative FiO_2_ and the incidence of PPCs in elderly non-cardiothoracic surgical patients.

However, this study has some important limitations must be mentioned. First, to reduce the risk of confounding factors, we adjusted for a large number of different risk factors and performed several sensitivity analyses, including propensity scoring. Second, residual and unmeasured potential confounding factors cannot be completely ruled out in observational studies. Third, patients with chronic lung diseases or underwent chest surgery were not included due to the potential influence on the outcomes of our study and the nature of the retrospective study. In addition, the results were derived from a single-center study; thus, the generalizability of our findings may be limited to other centers. Future randomized controlled trials are needed to confirm our results.

## 5 Conclusion

In this analysis of administrative data, a high intraoperative FiO_2_ (>60%) was associated with the postoperative occurrence of pulmonary complications independent of predefined risk factors in elderly non-cardiothoracic surgery patients. This finding was robust in a series of sensitivity analyses, including PSM and IPTW.

The preprint of this manuscript entitled *Higher fraction of inspired oxygen during anesthesia increase the risk of postoperative pulmonary complications in patients undergoing* non-cardiothoracic *surgery: A retrospective cohort study* is available at *doi. org/10.21203/rs.3.rs-4286848/v1*.

## Data Availability

The data analyzed in this study is subject to the following licenses/restrictions: The datasets generated and/or analysed during the current study are not publicly available as individual privacy could be compromised but are available from the corresponding authors on reasonable request. Requests to access these datasets should be directed to CZ, powerzcs@126.com.
